# Neurophysiological Correlates of Laboratory-Induced Aggression in Young Men with and without a History of Violence

**DOI:** 10.1371/journal.pone.0022599

**Published:** 2011-07-21

**Authors:** Daniel Wiswede, Svenja Taubner, Thomas F. Münte, Gerhard Roth, Daniel Strüber, Klaus Wahl, Ulrike M. Krämer

**Affiliations:** 1 Department of General Psychology II, Friedrich-Schiller-Universität, Jena, Germany; 2 Department of Psychology, Universität Kassel, Kassel, Germany; 3 Department of Neurology, Universität zu Lübeck, Lübeck, Germany; 4 Brain Research Institute, Universität Bremen, Bremen, Germany; 5 Department of Experimental Psychology, Carl von Ossietzky Universität Oldenburg, Oldenburg, Germany; 6 Psychosocial Analyses and Prevention - Information System, Munich, Germany; The University of South Wales, Australia

## Abstract

In order to further understand the mechanisms involved in planning an aggressive act, we conducted an event-related potential (ERP) study of young men with and without a history of violence. Participants completed a competitive reaction time task (based on the Taylor aggression paradigm) against a virtual opponent. In "passive" blocks, participants were punished by the opponent when losing the trial but could not punish, when winning, whereas in "active" blocks, participants were able to punish the opponent when winning, but were not punished when losing. Participants selected punishment strength in a decision phase prior to each reaction time task and were informed whether they had won or lost in the outcome phase. Additionally, a flanker task was conducted to assess basic performance monitoring. Violent participants selected stronger punishments, especially in "active" blocks. During the decision phase, a frontal P200 was more pronounced for violent participants, whereas non-violent participants showed an enhanced frontal negativity around 300 ms. The P200 might reflect the decision to approach the opponent at a very early state, the latter negativity could reflect inhibition processes, leading to a more considerate reaction in non-violent participants. During the outcome phase, a Feedback-Related Negativity was seen in both groups. This effect was most pronounced when losing entailed a subsequent inability to retaliate. The groups did not differ in the flanker task, indicating intact basic performance monitoring. Our data suggest that the planning of an aggressive act is associated with distinct brain activity and that such activity is differentially represented in violent and non-violent individuals.

## Introduction

Aggression and violence represent a major problem to society. The present study aimed to delineate neural correlates of aggression in violent and non-violent adolescents in order to elucidate whether violent participants showed measurable differences in brain response when aggression was experimentally induced.

Aggression is often subdivided into proactive and reactive subtypes [1], [2]. While proactive aggression is planned and goal-directed, reactive aggression is enacted instantly as a direct response to provocation and is therefore not inappropriate per se [1]. Reactively aggressive participants have been shown to be hyper-responsive to actual provocation [3]. Functional imaging studies point to aberrant interactions between frontal and limbic structures in individuals with histories of violence: While pre- and orbitofrontal structures show decreased activation, amygdala activation is increased in participants with histories of reactive aggression [2], [4], [5], [6].

The Taylor Aggression Paradigm (TAP) provides an established method to study aggression in the laboratory [7]. The TAP is a competitive reaction time task in which the participant competes against an opponent. In case of winning, the participant is asked to punish the opponent. In case of losing, the participant is punished by the opponent. Aggression in the TAP is most often operationalized as the mean punishment strength a participant selects for the opponent. Punishment strength selected in the first trial has been used as a measure of unprovoked aggression prior to the first interaction with the opponent [8], [9], [10], and the proportion of highest punishment selections has been used as an index of “extreme aggression” [8], [10]. It is a well-established finding that higher punishments are selected by aggressive men [8], [11], by participants lower in executive functions [12], by participants after provocation [12], [13], [14] and by participants high in trait aggressiveness [13].

Conclusions that can be derived from behavioral measures in the TAP are limited, as different cognitive, emotional and motivational processes can lead to similar behavioral output [15]. EEG- or fMRI-studies on aggression can help to reveal differences in underlying neural processes and thereby deepen our understanding and ultimately improve predictions of aggressive behavior. There are a few studies that combined laboratory-induced aggression and measurement of brain responses using the TAP [13], [14], [16], [17], [18], [19]. Krämer and colleagues [13] conducted an ERP study in which participants who scored either high or low on a trait aggressiveness scale performed a modified TAP. They played against two block-wise alternating fictitious opponents, who showed either fair (low provocation) or unfair (high provocation) behavior. The authors distinguished between a decision phase, where participants selected the punishment of the opponent, and an outcome phase, where participants were informed whether they had won or lost, and the opponents or the participants were punished accordingly. In the decision phase, Krämer et al. [13] reported an enhanced frontal negativity in high provocation blocks in high trait aggressive participants only - a component that the authors labeled “ Decision Related Negativity” (DRN). The DRN was most pronounced in high trait aggressive participants who actually behaved less aggressively during the experiment - indicating that the DRN could reflect “the neural correlate of aggression-controlling executive processes” [13] (p.1474). In the outcome phase, the authors reported an increased frontocentral negativity for “lost” compared to “won” feedback, which was identified as a “Feedback Related Negativity” (FRN) known from previous studies [20], [21], [22]. In a later EEG-study [17], the same authors used spectral decomposition of the data to extend and support their finding of frontal activity during decision-making and feedback evaluation, which was inversely related to the participant's experimentally induced aggressive behavior.

In the present study, we sought to investigate aggressive interactions in violent and non-violent participants (as defined by their prior history of aggressive behavior) using a modified version of the Krämer et al. [13] experiment. Modifications were made to punishment settings. It was assumed that aggressive behavior is more pronounced if there are no immediate consequences, and that participants are more able to control aggressive tendencies when aggression is punished. Aggressive behavior with and without immediate consequences was therefore incorporated by alternating blocks with inverse punishment/receiving punishment settings. Half of the blocks were “passive”, in which the subject was punished with an aversive tone when losing the trial, but could not punish the opponent when winning the trial. This pattern was reversed in “active blocks” in which the participant was not punished in the event of losing the trial, but could punish the opponent when winning the trial. In short, although participants were always required to select punishment strength in the decision phase, a punishment was received only when losing the trial in “passive” blocks, whereas the subject could punish the opponent when winning in “active” blocks. Another modification concerned the “behavior” of the virtual opponent. ERPs for high trait aggressive participants in Krämer et al. [13] were differentiated under conditions of high provocation. In the present study, participants played against one opponent only, and the provocation level was held high with participants losing 2/3 of the trials and relatively high punishment selections by the opponent.

At the behavioral level, we expected violent participants to behave more aggressively than control participants as indicated by higher mean punishment selection, higher first-trial punishment selection and a higher proportion of highest punishment level. It was predicted that such findings would be particularly apparent in “active” blocks when participants were able to retaliate for punishments received in previous blocks without the possibility of immediate consequences. For ERPs, it was predicted that a clear DRN would be seen in control participants, given their supposed ability to inhibit aggressive impulses. Because withholding aggressive impulses is only meaningful when the opponent can actually receive punishment, the DRN modulation was expected to be restricted to “active” blocks. In contrast, no modulation of the DRN was expected in the violent participants, as it was supposed that they would not inhibit their aggression. During the outcome phase, an FRN was expected for loss trials. In line with Krämer et al. [13], we anticipated a FRN-like component after win-trials for non-violent control participants, reflecting the negative valence of punishing someone else.

In order to have an estimate of “basic” executive functioning, participants completed an Eriksen flanker task known to assess action-monitoring processes [23]. Errors in such a task are reflected by a frontocentral negativity known as Error Related Negativity (ERN; [24], [25]. Previous research reports no ERN malfunctions in psychopathic violent offenders in a standard flanker task [26], [27]. However, there is unclear evidence whether the error positivity (P_e_) [25], a component following the ERN around 200 to 400 ms after error commission, is reduced in those subjects. Among other theoretical accounts, there is the idea that the P_e_ might reflect motivational significance or conscious error processing [28], [29]. One study reports reduced P_e_ amplitude [26] for psychopaths, whereas another study [27] does not provide clear evidence. It was suggested that the P_e_ reduction might be found in individuals with psychopathy only, but not in violent subjects who do not meet the criteria for psychopathy [26].

## Results

### Aggression Paradigm

#### Questionnaire and Behavioral Data

**Figure 1:**
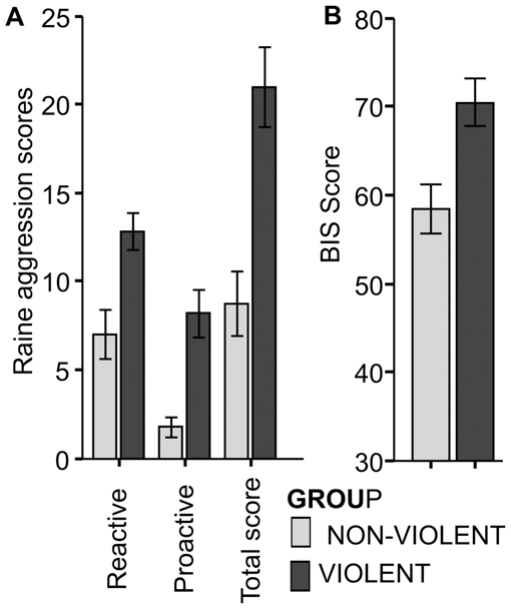
Results of Questionnaires. a) Total aggression score from the Reactive-Proactive- Aggression Questionnaire (RBQ) b) Total scores for impulsivity from the Barratt-Impulsiveness Questionnaire (BIS-11).

The Psychopathic Personality Inventory-Revised (PPI-R) composite score as a measure of psychopathic traits showed higher scores for participants of the violent group than for controls (t(15)  = 2.5; p<.02; η^2^ = .3; non- violent group: mean 320; SD 28; range 256–346; violent group: mean 365; SD 45; range 271–401). Subjects of the violent group scored also higher on the Reactive-Proactive-Aggression Questionnaire (RPQ) on both proactive and reactive aggression scales (RPQ; proactive, reactive, sum score, t (18) >3.4; p<.003, η^2^ (sum score RPQ)  = .47, see Figure 1a). Violent participants were more impulsive as indicated by a higher Barratt Impulsiveness Scale (BIS-11) score (t(18)  = 3.0; p<.01 , η^2^ = .34 ,see Figure 1b). Participants scoring high on the aggression questionnaire scored also high on impulsivity scores (correlation BIS-11 and RPQ total score, r = .66; p<.01).

**Figure 2:**
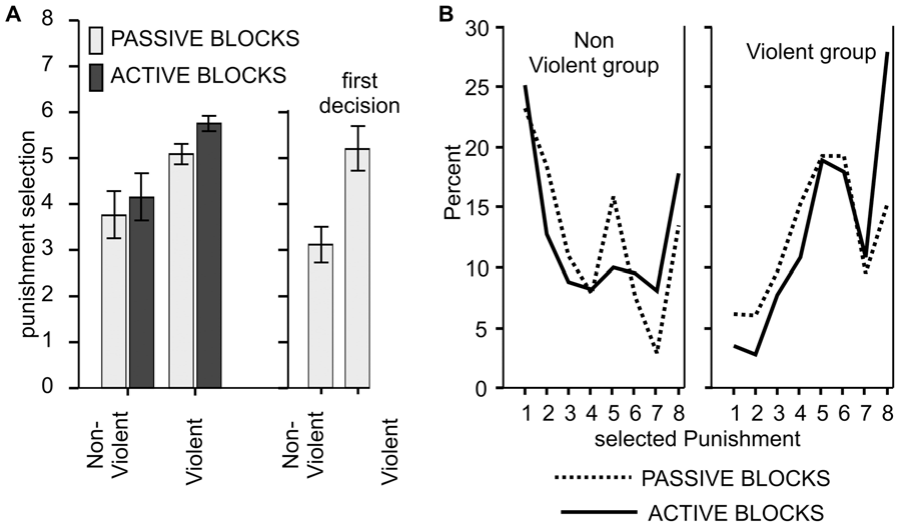
Punishment strengths. a) Mean punishment strengths selected in the decision phase. The rightmost bars of Figure 2a depict the first selection at the beginning of the experiment. b) Percentage of punishment strength 1 to 8 given during the experiment.

Behavior data in the TAP showed that violent participants selected stronger punishments for their opponents (Figure 2a), and both groups selected higher punishment levels in “active” blocks (ANOVA based on mean punishment selection in the TAP; BLOCK: F(1,18)  = 14.5; p<.001; GROUP: (F(1,18)  = 8.9; p<.008, η^2^ = .43; Interaction n.s.). A preference for higher punishment levels in the “active” blocks was found in all violent participants and in eight of the nine non-violent participants. Subjects with high RPQ total scores selected higher punishments in “active” as well as in “passive” blocks (correlation RPQ/mean punishment selection: “passive” blocks: r = .54; p<.01, “active” blocks: r = .59; p<.001). Mean punishment strength correlated positively with RPQ total scores (r = .59; p<.006) and the BIS-11 score (r = .45; p<.049).Participants in the violent group selected also higher punishment levels at the first trial (first trial punishment selection; t(18)  = 3.4; p<.003, η^2^ = .039; see Figure 2a).

As depicted in Figure 2b, both groups differed in the proportion of lowest and highest punishment selection. Non-violent participants selected the minimal punishment level most often (23.2% of all selections in “passive” blocks, 25.1% in “active” blocks), whereas the violent participants chose the mildest punishment infrequently (ANOVA based on percentage of punishment selection 1; GROUP: F(1,18)  = 5.3; p<.034, η^2^ = .23; BLOCK and interaction n.s.). In contrast, the highest punishment level 8 was preferably given in “active” blocks. Although this tendency was numerically more pronounced in the violent group, the BLOCK x GROUP interaction failed to reach significance (ANOVA based on percentage of punishment selection 8; BLOCK F(1,18)  = 10.2, p<.005, η^2^ = .33; BLOCK x GROUP: F(1,18)  = 2.4; p<.14, η^2^ = .08; GROUP n.s.).

Reaction times indicated faster responses to the visual target stimulus in “active” relative to “passive” blocks (RTs in ms, “active” blocks: Non-violent 135, violent group 131; “passive” blocks: Non-violent 143, violent group 154). Although this pattern was seen in both groups, there was a tendency for greater differences between “passive” and “active” blocks in the violent group (ANOVA based on reaction times, BLOCK F(1,18)  = 13.5; p<.001, η^2^ = .38; GROUP n.s.; Interaction BLOCK x GROUP: F(1,18)  = 3.7; p<.07, η^2^ = .1; t-tests separately for both groups: violent group: t(10)  = 3.4; p<.01; non-violent group: t (8)  = 1.8; p<.1; n.s.).

#### ERPs in the Decision Phase

**Figure 3:**
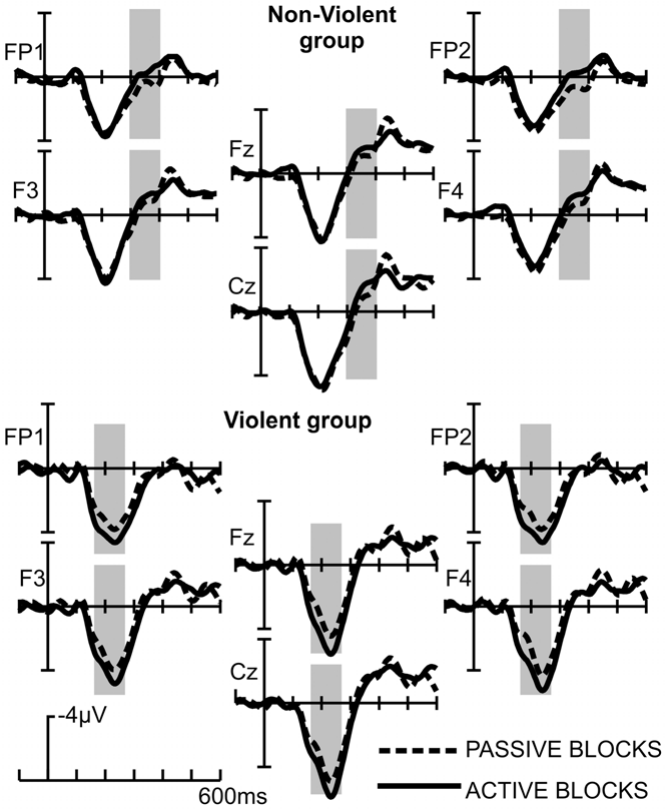
ERPs in the decision phase. Depicted are frontocentral electrodes locked to the onset of a question mark. Participants had the task to decide for a punishment. Grey-shaded areas indicate time windows for statistical analysis.

ERPs differed as a function of “passive/active” blocks and group in two time regions (Figure 3). Firstly, enhanced positivity was detected during “active” blocks in the violent group between 150 and 250 ms in frontal, central and parietal electrodes; the positivity was most pronounced on fronto-central electrodes. No difference between “active” and “passive” blocks in the non-violent group was detected during this time-window. We refer to this effect henceforth as “early positivity”. Subsequently, a negativity was detected in both groups and blocks between 300 and 400 ms - an effect observed to be most pronounced in non-violent participants in “active” blocks. This effect was strongest on frontal and frontopolar electrodes. In line with Krämer et al. [13], this deflection is referred to as “Decision-Related Negativity” (DRN).

Statistical analysis confirmed the presence of early positivity, yielding a significant BLOCK x GROUP interaction ((F(1,18)  = 9.9; p<.006, η^2^ = .31), separate t-tests for both groups on all 4 electrodes; violent group: t(10) <−2.4; p<.03; non-violent group: t(8) max (2.8) min (−.4); p>.07).

Differences in the DRN were also confirmed statistically by a significant BLOCK x GROUP interaction (F(1,18)  = 5.3; p<.03, η^2^ = .21). Post-hoc analysis confirmed more negative ERPs in the “active” blocks for non-violent participants only (non-violent: t(8) >2.2, p<.05; violent: t(10) <.7; p>.4; n.s. see Figure 3). There were no further differences found in time windows later than the DRN.

#### ERPs in the Outcome Phase

**Figure 4:**
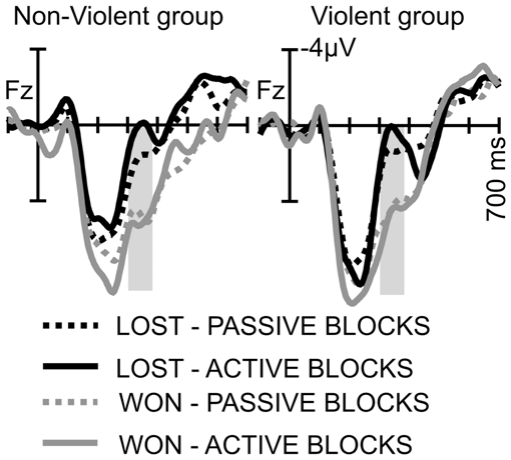
ERPs locked to the onset of the Feedback screen. ERPs are separated by blocks (“passive” and “active”; solid vs. dotted lines) and outcome (lost and won, black vs. gray line). Gray-shaded areas indicate time window of the FRN.

The outcome phase was characterized by the “Feedback Related Negativity” (FRN), which was superimposed on a large positive deflection (referred to as P3) and more pronounced in loss trials (Figure 4). It was seen in both groups (ANOVA, Factor OUTCOME; F(1,18)  = 17.4; p<.001, η^2^ = .49), and there was a tendency for the FRN to be more negative for loss trials in “active” blocks (OUTCOME x BLOCK; F(1,18)  = 3.25; p<.088, η^2^ = .15, no further significant main effects or interactions).

### Flanker task

#### Behavioral Data

As expected, participants were faster on incorrect relative to correct responses and faster in congruent relative to incongruent flanker trials. There were no group differences in response time (ANOVA, ACCURACY: F(1,18)  = 108.4; p<.001, η^2^ = .86; CONGRUENCY (congruent vs. incongruent flanker stimuli): F(1,18)  = 29.0; p<.001, η^2^ = .61; GROUP: F(1,18) <1; no significant interactions) or in error rates (ANOVA on percentage of erroneous responses, CONGRUENCY: F(1,18)  = 101.8; p<.001, η^2^ = .85; GROUP: F(1,18) <1, no significant CONGRUENCY x GROUP interaction). There were no significant correlations between BIS-11 scores and reaction time measures (RT errors, RT correct, RT differences error and correct). Reaction times did not correlate significantly with TAP measures.

#### Response-locked ERPs

**Figure 5:**
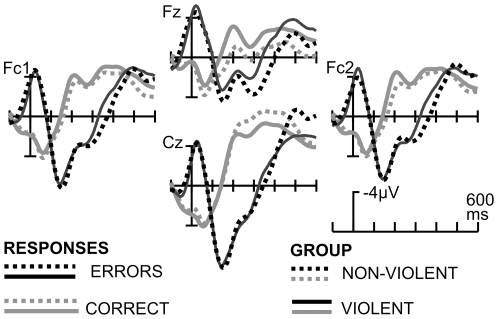
ERPs in the flanker task. ERPs are locked to the response; correct responses are shown in gray scale, erroneous responses are shown in black. Groups are indicated by line style.

There was a clear ERN following erroneous responses when compared with correct responses (Figure 5). Visual inspection showed no differences between groups. This was confirmed by statistical analysis (ANOVA with ACCURACY, ELECTRODES and GROUP as factors; ACCURACY F(1,18)  =  51.9; p<0.01, η^2^ = .73 ; GROUP, ELECTRODES and interactions: F<1. After the ERN, there was a clear positive component, which will be referred to as P_e_. Visual inspection might suggest group differences in the P_e_ starting around 250 ms after the error, but group differences were not confirmed statistically (ANOVA as above, ACCURACY F(1,18)  = 99.5; p<.001, η^2^ = .84; GROUP x ACCURACY and GROUP x ACCURACY x ELECTRODES n.s.). Statistics conducted as in [26] based on difference waves on electrode Cz did not change this pattern. ERN and P_e_ amplitudes and difference wave (error minus correct) did not correlate significantly with BIS-11 or RPQ-scores or with TAP measures.

## Discussion

### Summary of results

The present study used a modified Taylor Aggression Paradigm to investigate behavioral and neurophysiological differences between participants with and without a history of violence. Both violent and non-violent participants selected stronger punishments when they believed that the punishment would be actually delivered to the opponent. ERPs in the decision phase showed a relatively early frontal positive ERP deflection which was most pronounced for violent participants when allowed to punish. Non-violent participants showed a somewhat later frontal negativity in “active” trials when allowed to punish. In addition, a flanker task was conducted to examine whether the tendency to react aggressively was related to differences in basic executive functioning as has been suggested by some authors [12], [30], [31]. However, differences between violent and non-violent participants emerged neither behaviorally nor electrophysiologically in this task. Differences to previous research, who reported slower reaction times [26], [27] and increased P_e_ amplitude [26], might either be attributed to the higher level of psychopathic traits [26] or to limited power due to our small sample size. Taken together, results in the flanker task suggest that aggressive tendencies in the violent group are not driven by performance monitoring deficits. We will thus only discuss results from the Taylor Aggression Paradigm in detail.

### Behavioral Data

Questionnaire data clearly differentiated both groups: Participants with a history of violence scored higher on scales for proactive and reactive aggression, psychopathic traits and impulsivity. Although participants of the violent group scored higher on impulsivity than controls, their impulsivity score was well beyond the scored reported for male offenders in a former study [32]. There were some individuals in the violent group scoring very high on psychopathic traits whereas some did not differ from the control group. Thus, the violent group contains both individuals with and without strong psychopathic traits. This supports recent research [33] pointing out that psychopathic traits do not represent a discrete class among youthful offenders, they are best characterized as a continuum.

In the TAP, violent participants selected higher punishments for the opponent. Even without initial provocation, violent participants selected higher punishment in the first trial, which could indicate a dispositional tendency to act aggressively. Moreover, participants with high aggression and impulsivity scores on questionnaire measures selected higher punishments during the decision phase. Taken together, our results provide further evidence for the validity of the TAP [8] and its feasibility for examining aggressive behavior in laboratory settings. Both groups selected higher punishments when they were under the impression that the punishment would be executed (“active” blocks). Thus, both groups reacted to provocation with increased aggressiveness in “active” blocks, but punishment selections were overall higher in violent participants. Previous suggestions that impulsive behavior results in shorter reaction times [34] are not supported by the present study. In addition, behavioral measures of impulsivity did not covary with self-reported impulsivity, replicating previous findings [12], [35] and suggesting that these measures refer to different aspects of impulsivity [12].

### ERP findings

#### Decision phase

The finding of an early increased positivity in the violent participants during the decision phase was unexpected since Krämer et al. [13] did not report group differences for this frontocentral positivity. However, differences in design between the current study and Krämer et al. [13] could underlie these differences. Firstly, the block-wise change between active and passive roles with respect to punishment was a feature only of the current study. Moreover, and more likely to explain differences, were issues related to the sample. In the study by Krämer et al. [13], participants were not selected based on past aggressive acts but from a student sample on the basis of trait aggressiveness determined by a questionnaire. The observed positivity might therefore be specific to highly aggressive participants.

One positive component recently discussed in relation to aggression is the P3a [34]. This component is related to novelty processing and has been linked to frontal lobe engagement and attentional mechanisms [36]. However, the early positivity observed in the current study had a shorter latency than the typical P3a. We therefore assume that the early frontal positivity is an instance of the P200 and not a P3a - which it might be mistaken for [37]. The P200 is an attention-related component with an onset at around 150 to 200 ms, modulated by emotional and motivational significance of a stimulus. Changes in P200 amplitude have been associated with greater mobilization of attentional resources by negative pictures [38] or threat-related words [39]. More similar to the present study, Bertsch et al. [14] used a TAP task with a high and low provocation group of healthy subjects. They report increased P200 amplitude in the provoked relative to the non-provoked group in response to emotional pictures. Bertsch et al. [14] suggested that experimentally induced aggression might alter early global affective evaluation or categorization processes. Here we show that this global affective processing is not restricted to the presentation of emotional stimuli (masked faces in [14]). Instead, the increased P200 after provocation can also be found when deciding for a punishment. This supports the idea that the P200 might be critical for subsequent approach and withdrawal behavior [40] and might reflect the decision to approach the opponent at a very early state. However, in the present study, the P200 increase was restricted to aggressive subjects when allowed to punish. This might also be explained within the P200 framework, since P200 enlargement has also been shown for potentially dangerous or high-valence stimuli [37], [38], [41] and when identifying risky situations [42]. Further, the P200 has been shown to be larger for gains than for losses in a monetary gambling task [37]. One might therefore speculate that selection of a punishment in “active” blocks is regarded as a reward for violent participants, but not for non-violent participants. This would be in line with neuroimaging results of Krämer et al. [16], which demonstrated increased activity in the dorsal striatum during the decision to retaliate, possibly related to reward expectancy.

The second difference between both groups was an increased frontal negativity for non-violent participants in “active” blocks. The effect reported in the current study was very similar to the DRN reported by Krämer et al. [13]. The DRN cannot be attributed to motor responses, since the decision phase did not require any motor-related action. Krämer et al. reported that high trait aggressive participants, who nevertheless behaved non-aggressively, showed the strongest DRN. The authors of this study therefore suggested that the DRN reflects general monitoring or inhibition processes driven by a conflict between a wish to retaliate on the one hand and to prevent escalation on the other. Thus, we might expect the DRN to be smaller or absent when the opponent cannot be punished, as was the case in the “passive” blocks of the current study. In line with Krämer et al., we suggest that the lack of a DRN modulation in the violent participants is due to impaired self-regulation processes.

#### Outcome Phase

The Outcome Phase was characterized by an FRN after lost trials, which is in line with previous research [20], [22], [43] and might reflect the motivational value of losing a trial [44]. Interestingly, there was a tendency for a more negative FRN in “active” blocks. At first glance, this is surprising, since losing in “active” blocks did not result in punishment, whereas losing in “passive” blocks was associated with an unpleasant sound. Event evaluation along a positive-negative dimension [43] would entail that losing with punishment expectation (“passive” blocks) was more negative than losing without punishment expectation (“active” blocks). Thus, a more negative FRN for “passive” blocks would be expected, which is the opposite of what we obtained in the current study. However, losing a trial in “active” blocks also entailed losing the chance to retaliate, whereas there was never such a chance in “passive” blocks. Thus, the pattern of results could suggest that missing an opportunity to retaliate is more of a punishment than receiving a punishment itself. Most interestingly, both groups did not differ in this pattern, indicating that missing a chance to punish an unfair opponent had a high motivational value for both violent and non-violent participants.

Winning, and thus being able to punish, did not elicit FRN-like components for either violent or non-violent participants. This is surprising, particularly as Krämer et al. reported an FRN-like component for non-aggressive participants after winning. This, they speculated, might indicate that winning (and thus being able to punish the opponent) was perceived as a negative event in these participants. Indeed, an FRN has also been shown in experiments that required participants to observe the consequences of their actions for others [45], [46], [47], [48]. The lack of a similar effect in the present study could be due to several reasons - most notably, differences in sample characteristics. Whereas the present study only comprised young men, the Krämer et al. sample also included women, for whom a greater sensitivity of the FRN to another person's loss has been reported [49]. Further, participants of the current experiment lost two thirds of the trials, which might have diminished empathic responses for the one third of trials in which the opponent lost.

One shortcoming of the present study is the rather small sample size. This problem is often faced in studies with special demands on the subject group and limits the power to detect small effects. On the other hand, very strict statistical testing including correction for multiple comparisons might lead to false negativities. Thus, definite conclusions should not be made until the findings have been replicated in larger samples. Second, we report no differences between groups in basic executive processes. It should be noted, however, that executive processes also include working memory, behavioral inhibition, strategic planning and other functions which were not examined in the present study and which might be impaired in violent participants. In addition, ERP recordings were conducted using the left mastoid reference, which precludes conclusions about the laterality of effects. Future studies should also use linked mastoid or average reference.

To conclude, the present study showed both behavioral and neurophysiological differences between violent and non-violent young men in a laboratory aggression task. Violent and non-violent participants did not differ in basic performance monitoring processes . This suggests that their violent tendencies and lack of self-regulation in a social interaction are not caused by a general deficit in executive functioning. ERPs related to the decision to retaliate indicated a stronger attentional allocation as well as reduced frontal control in violent participants. The observed ERP differences were subtle however, suggesting that there are no fundamental neurobiological malfunctions in our sample of violent participants. Thus, we demonstrate that further understanding of the neural correlates of aggression is to be gained from experiments more directly related to aggressive behavior.

## Materials and Methods

### Participants

Twenty young men participated in the study; eleven of whom reported a history of violent behavior. The violent group was recruited with the help of local street workers and a counseling centre for victim-offender mediation in the city of Bremen. Inclusion criteria were as follows: At least one conviction for violent offence, no incarceration, age between 18 and 25 years, male gender and German nationality. The majority of the violent group had been regularly involved in committing physical assaults. The non-violent group was recruited from a local school and matched for age, sex and IQ with the violent group. The mean age was 20.5 years for both groups, ranging from 18 to 24 in the violent group and 18 to 25 in the non-violent group. Exclusion criteria for both groups were insufficient knowledge of the German language, neurological impairments, substance abuse and psychosis. None of the participants has been in psychological or psychiatric treatment during the time of the examination. After completion of the EEG experiment and a set of questionnaires and interviews (conducted in extra meetings), participants received € 80 for participation. All participants had normal or corrected to normal vision and provided written informed consent according to the Declaration of Helsinki. The experiment was approved by the University of Magdeburg ethics committee (affiliation of DW, UMK and TFM at the time of experimentation).

### Measures

#### Psychopathic traits

Psychopathic traits were captured with the German version of the Psychopathic Personality Inventory-Revised (PPI-R)[50]. The PPI-R is a 154 items questionnaire that yields 8 subscales on the two-factor structure “Fearless Dominance” and “Impulsive Antisociality”. Since the two factor structure has recently been called into question [51], we used the composite score in the current analyses. The PPI-R has been well validated for use on both offender [52] and community [53] samples. It does not provide cut-off-scores [54].

#### Aggression

The profile and degree of proactive and reactive aggression was assessed using the Reactive-Proactive-Aggression Questionnaire (RPQ; [55] which comprises 23 items scored between 0 (never) and 2 (often). Items are summed to form a total score and load on two subscales, proactive and reactive aggression. The higher the scores the more aggressive behavior is reported, the RPQ does not provide cut-off-scores. The TAP provides several behavior measures for aggressive behavior. Mean punishment strength selected for the “opponent” [7] was used as an index for overall aggressiveness, while first trial punishment strength was used as a measure of unprovoked aggression [8], [9], [10] The proportion of highest punishment selection served as an index of extreme aggressiveness [8], [10] and the proportion of lowest punishment selection as an index of participants' refusal to punish harshly [10].

#### Impulsivity

Impulsivity was measured with the German version of the Barratt Impulsiveness Scale (BIS-11) [56]. The BIS-11 is a 24-item self-report questionnaire requiring assessment on a four-point scale from “rarely/never” to “always”. The sum of all items constitutes the total score [32]. Scores range from 24–96 with higher scores indicating greater impulsivity. The BIS-11 does not provide cut-off values, on average, male offenders score 76.3 [32].

Two behavioral measures from the TAP were used to examine impulsivity: a) Premature responses, defined as the total number of responses preceding the actual stimulus, b) Response time following target stimulus. The procedure allowed recording of negative response times which were used as a measure of impulsivity and defined by the time the response preceded the actual stimulus.

In addition, reaction times and error rates to flanker stimuli in the flanker task were used to provide measures of impulsivity in a paradigm without a social component (no opponent, no punishment).

### Task and Procedure

Participants were interviewed, completed questionnaires and participated in the EEG-experiments in three separate testing sessions (results from the interviews will be reported elsewhere).

#### Aggression paradigm

The EEG experiment was programmed using Presentation software (www.neurobs.com) and presented on a standard PC. The experiment was a modified version of the Taylor Aggression Paradigm [7], a version of which has been used previously in an EEG study of our group without the role change between experimental blocks [13].

Participants were instructed that they would be playing a reaction-time task against another young man, also in an EEG environment, in another room in the building, and that they will receive or administer an unpleasant tone as a punishment under some circumstances. The opponent, who was actually a confederate of the experimenters, was introduced prior to EEG setup procedure. Both young men listened jointly to the instructions. After instruction was given to both players, one of the experimenters escorted the opponent/confederate out of the laboratory to “guide him to the second lab, where another team of experimenters [was] waiting for the setup procedure”.

**Figure 6:**
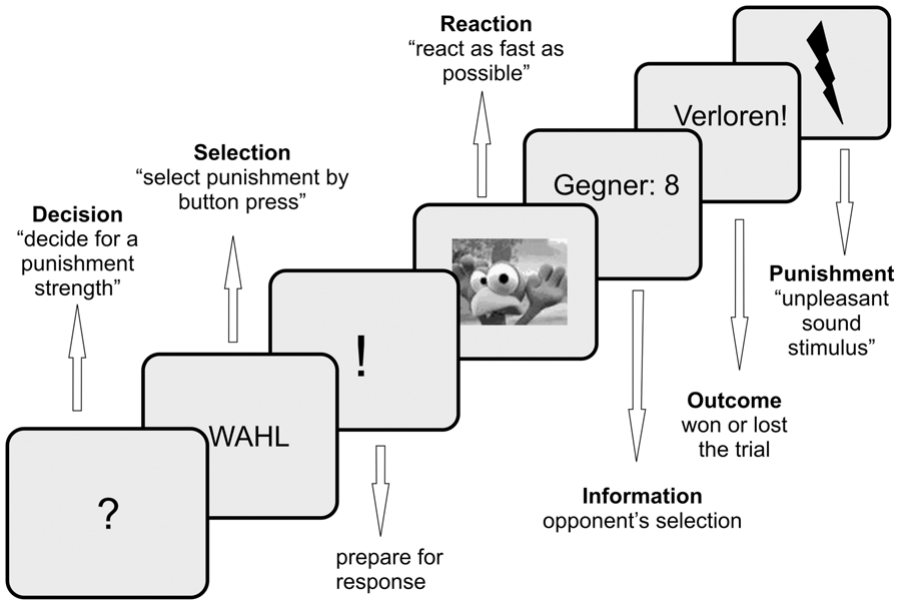
Experimental procedure. Participants were told that they are playing against another young man, who was introduced prior to the experiment. Trial structure: There was a Decision phase, a Selection Phase, a Reaction Phase and an Outcome phase in each trial. In the decision phase, participants selected the punishment (strength 1 to 8) which the opponent receives in case of losing the trial. Selection was followed by a “!” screen with variable interval between 600 and 800 ms. In the subsequent reaction phase, participants were instructed to respond as fast as possible to the appearance of a comic chicken. In the outcome phase, feedback was given whether the participant won (faster reaction to the chicken) or lost the trial. 40 trials constitute a block. There were alternating “passive” and “active” blocks. In “passive” blocks, the participant received an unpleasant sound stimulus at the end of the trial when they had a lower reaction time than the opponent, whereas the opponent did not receive a punishment when losing the trial. This pattern was reversed in the “active” blocks: The participant did not receive the punishment in case of loss, but the (virtual) opponent received the unpleasant sound stimulus; stimulus strength was determined by the participant in the decision phase. See text for further explanations.

Experimental trials: The experiment comprised eight blocks with 40 trials each. Every trial commenced with a decision phase, indicated by a question mark shown for 1.5 seconds. Participants were instructed to consider a punishment administered to the opponent in the case that the opponent lost the upcoming trial. A screen with the German word for “selection” followed, which required the participant to select the punishment (selection phase). Punishment selection was done by button press on the keyboard, with key 1 indicating the mildest and key 8 indicating the strongest punishment. Punishment selection was followed by a fixation period ("!" was presented in the center of a monitor for a variable time interval ranging from 600 to 800 ms) to prepare for the upcoming response. In the subsequent reaction phase, participants were instructed to press the mouse button as soon as a visual prompt (a well-known bird from a computer game) appeared on the screen. The reaction phase ended by a button press and was followed by a screen displaying the opponent's punishment selection for 1.5 seconds (information phase). Subsequently, a screen indicated whether the participant had won or lost the trial by presenting the German word for “won” or “lost” for 1 second. The trial ended with punishment administration, which differed block-wise (Figure 6).

Experimental blocks: “Passive” and “active” blocks were alternated, each comprising 40 trials, with the first block always being “passive”. Trials in “passive” and “active” blocks differed in the way the punishment was administered. In “passive” blocks, the participant was punished whenever he lost the trial (participants' reaction time in the reaction phase was determined to be slower than the reaction time of the virtual opponent). Punishment was given via administration of an unpleasant polystyrene scratching noise, administered in 8 different volumes, ranging from mild to very loud. The volume of the unpleasant tone reflected the opponent's punishment selection and matched the numerical value given in the information phase. During the “passive” blocks, participants knew that the opponent was not punished when the opponent lost a trial. However, participants were still required to select punishment strength in the decision phase and were informed that selected punishment strength served as a threat for the opponent. The punishment pattern was reversed in “active” blocks: The opponent, but not the participant, received the punishment when the opponent lost the trial. Punishment intensity was determined by the button press response in the selection phase. In short, at the end of each trial in “passive” blocks, the participant was punished for losing, while the opponent was not punished. In “active” blocks, participants were not punished when losing, while the opponent was punished if the participant won the trial. Unpleasant tones in the punishment phase were administered via multimedia speakers close to the presentation monitor.

Frequency of wins and losses and the opponent's selection were under full experimental control. It was intended that each participant would experience a loss rate of 2/3 over all trials. However, to increase plausibility that participants were playing against a real opponent, all reactions above 500 ms, all omitted responses and all premature responses resulted in losing the trial. To compensate for the additional losses, the *a priori* loss rate was set to 64 percent, with the actual loss rate being 68 percent on average. There were no differences in frequency of win and loss feedback between both groups. However, due to increased number of premature responses in “active” blocks, there was a slight increase of loss trials in “active” blocks (67% loss trials in “passive” vs. 69% in “active” blocks). This holds true for both groups. Punishment intensity administered to the participants was selected by a partly adaptive algorithm: In 75% of the trials, the program selected a punishment between 3 and 8; in the remaining 25% of trials, the algorithm mirrored the punishment strength selected by the participant in the previous trial. Although this algorithm leads to slight differences in the punishments administered to the participants (i.e. giving higher punishment levels results in receiving higher punishment levels), this combination of static and adaptive punishment selections increased the plausibility that participants were playing against a real opponent. In fact, open questions after the experiment revealed that all of the participants believed they had been playing against a real opponent.

Volume of the tones was adjusted prior to the experiment so that participants judged the loudest tone to be unpleasant, but not harmful using a special purpose program with the same scratch noises as in the actual experiment. Participants were completely debriefed after the end of the experiment.

#### Flanker task

An adaptation of the Eriksen-Flanker task [23] was used. Participants were instructed to respond as fast and as accurately as possible with the left index finger if the center letter of a 5 letter array was an H and with the right index finger, if the letter was an S. There were 60% congruent (HHHHH or SSSSS) and 40% incongruent (HHSHH or SSHSS) trials. Flanker stimuli consisted of black capital letters (‘Courier new’ font, in front of a gray background, stimuli covered 2.18° of visual angle). A single trial had the following sequence (timing is provided in brackets): fixation cross (600–800 ms, mean 700 ms), flanker stimulus until response. Participants could take a short break after every block (70 trials). In total, there were 1050 Flanker stimuli (15 blocks), the experiment lasted between 22 and 31 minutes.

### EEG Recording

The electroencephalogram (EEG) was recorded using a Schwarzer Amplifier. Signals were recorded from 27 positions including all 19 standard locations of the 10/20 system using tin electrodes mounted in an elastic cap (EasyCap). A left mastoid reference was used. Eye movements were recorded with electrodes affixed to the right and left external canthi [horizontal electrooculogram (hEOG), bipolar recording] and at the supra- and infraorbital ridges of the left eye [vertical electrooculogram (vEOG), bipolar recording]. Impedances of all electrodes were kept below 10 kOhm. Biosignals were amplified with a digitization rate of 250 Hz.

Prior to ERP analysis, all trials containing artifacts were discarded, using a special purpose program (ERPSS) with individualized peak-to-peak-amplitude criteria based on visual and semi- automatic inspection of vEOG, hEOG and head channels. In the aggression paradigm, eye artifacts for six participants with extensive blinks (four from the violent group) were corrected using blind component separation (SOBI) [57], which has been shown to be superior to other artifact correction procedures [58] . ERPs in the aggression task were averaged relative to a 200 ms baseline prior to stimulus onset. Stimulus-locked ERPs were generated separately for the decision phase (question mark stimulus; indicating that the participant is required to decide on a punishment for the opponent) and for the outcome phase (feedback screen; indicating whether the participant lost or won the trial).

ERPs in the flanker task were generated relative to a 100 ms pre-response baseline. Consistent with previous research [59], [60], [61], only responses given within 200–800 ms after the flanker stimulus onset were included in data analysis.

### Data Analysis

#### Aggression paradigm

Unless otherwise stated, behavior and ERP data were subjected to analysis of variance (ANOVA), containing the within factor BLOCK (“passive” vs. “active”) and the between factor GROUP (history of violence vs. no history). ERPs in the outcome phase included the additional factor OUTCOME (whether the participant won or lost the trial).

ERPs were analyzed separately for the decision and the outcome phase. *Decision phase:* Time windows of interest were defined by visual inspection and on the basis of prior results [13]. Widespread GROUP x BLOCK difference were observed between 150 to 250 ms (referred to as early positivity). Furthermore, a fronto-central difference in an 300 to 400 ms time window was apparent (Decision Related Negativity (DRN), see [13]. Mean amplitudes were quantified on electrodes FP1, FP2, F3 and F4 (factor ELECTRODES) in a 150–250 ms time window for the early positivity and a 300 to 400 ms time window for the DRN. *Outcome phase:* A frontocentral negativity emerged for lost vs. won trials akin to the feedback related negativity (FRN). As the FRN is known to have a fronto-central distribution, we conducted a similar analysis as performed by Krämer et al. [13] and subjected the mean amplitudes for a time window from 300 to 370 ms at electrode Fz to an ANOVA containing the factors BLOCK, GROUP and OUTCOME.

#### Flanker Task

Statistical analysis was based on the within factor ACCURACY (correct vs. erroneous responses) and the group factor GROUP (violent vs. non-violent participants). The ERN was examined at its topographical maximum at electrodes FC1, FC2, Fz, Cz (factor ELECTRODES) and was quantified by a peak amplitude measure in a time window 0 to 100 ms after the erroneous (ERN) or the correct response. The later positive component after error commission (P_e_) was analyzed with the same factors as the ERN based on mean amplitudes (250–400 ms after error commission); since it is known that the P_e_ has a somewhat more parietal distribution, analysis was conducted on electrodes Cp1, Cp2, Cz, Pz.

All ERP statistics in both experiments are based on unfiltered data (except band-pass from 0.5 to 70 Hz during recording); to remove high frequency noise, ERP figures are displayed with a 12 Hz low pass filter. ERP data were analyzed and displayed using a purpose tailored program (ERPSS); statistical analysis of behavior and ERP data was conducted with SPSS 15. Degrees of freedom are provided uncorrected; whenever necessary, p-values are Greenhouse-Geisser corrected to account for possible violations of the sphericity assumptions.
